# A neural circuit for gastric motility disorders driven by gastric dilation in mice

**DOI:** 10.3389/fnins.2023.1069198

**Published:** 2023-02-22

**Authors:** Xi-yang Wang, Xiao-qi Chen, Guo-quan Wang, Rong-lin Cai, Hao Wang, Hai-tao Wang, Xiao-qi Peng, Meng-ting Zhang, Shun Huang, Guo-ming Shen

**Affiliations:** ^1^School of Integrated Chinese and Western Medicine, Anhui University of Chinese Medicine, Hefei, Anhui, China; ^2^Research Institute of Acupuncture and Moxibustion, Anhui University of Chinese Medicine, Hefei, Anhui, China; ^3^School of Life Sciences and Medicine, University of Science and Technology of China, Hefei, Anhui, China; ^4^Institute of Integrated Chinese and Western Medicine, Anhui University of Chinese Medicine, Hefei, Anhui, China

**Keywords:** gastric motility disorders, neural circuit, paraventricular nucleus of the hypothalamus, dorsal motor nucleus of the vagus, corticotropin-releasing hormone

## Abstract

**Introduction:**

Symptoms of gastric motility disorders are common clinical manifestations of functional gastrointestinal disorders (FGIDs), and are triggered and exacerbated by stress, but the neural pathways underpinning them remain unclear.

**Methods:**

We set-up a mouse model by gastric dilation (GD) in which the gastric dynamics were assessed by installing strain gauges on the surface of the stomach. The neural pathway associated with gastric motility disorders was investigated by behavioral tests, electrophysiology, neural circuit tracing, and optogenetics and chemogenetics involving projections of the corticotropin-releasing hormone (CRH) from the paraventricular nucleus of the hypothalamus (PVN) to acetylcholine (ChAT) neurons in the dorsal motor nucleus of the vagus (DMV).

**Results:**

We found that GD induced gastric motility disorders were accompanied by activation of PVN*^CRH^* neurons, which could be alleviated by strategies that inhibits the activity of PVN*^CRH^* neurons. In addition, we identified a neural pathway in which PVN*^CRH^* neurons project into DMV*^ChAT^* neurons, modulated activity of the PVN*^CRH^*→DMV*^ChAT^* pathway to alleviate gastric motility disorders induced by GD.

**Discussion:**

These findings indicate that the PVN*^CRH^*→DMV*^ChAT^* pathway may mediate at least some aspects of GD related gastric motility, and provide new insights into the mechanisms by which somatic stimulation modulates the physiological functions of internal organs and systems.

## 1. Introduction

Functional gastrointestinal disorders (FGIDs) are widespread, and constitute a major personal and socio-economic burden (Drossman, 2016). A recent global epidemiological study (Sperber et al., 2021) has found that over 40% of the world’s population suffers from FGIDs. The disease is characterized by chronic abdominal discomfort without structural or biochemical causes, and its etiology and pathophysiology are multifactorial and still incompletely defined. Stress is widely relevant to the pathophysiology and treatment of digestive disorders. The latest Rome IV guidelines identify FGIDs as a disorder of gut–brain interaction (Drossman and Hasler, 2016), and stress contributes dynamically to various pathways of brain–gut communication, including the autonomic nervous system, the HPA axis, the local immune system, and brain mechanisms (Labanski et al., 2020). Changes in brain–gut interactions may underlie the symptoms of several FGIDs, including functional dyspepsia (FD) and irritable bowel syndrome (Mayer et al., 2006; Drossman, 2016).

The gastrointestinal tract and its enteric nervous system are innervated by the autonomic nervous system, which provides an efferent pathway for the stress-induced modulation of the gastrointestinal sensorimotor function, with the role of the vagus nerve in gastrointestinal sensitivity and motility having interesting clinical implications (Bonaz et al., 2018). From a neuro-gastrointestinal perspective, the functions of the upper gastrointestinal tract, including gastric tone and motility, are regulated by the activity of pacing neurons in the dorsal motor nucleus of the vagus (DMV), and its activity is regulated in turn by the tonic GABAergic input from the adjacent nucleus tractus solitarius (NTS) (Travagli and Anselmi, 2016) as well as inputs from higher centers, including projections from the paraventricular nucleus of the hypothalamus (PVN) (Browning and Travagli, 2014). PVN is an important autonomous control center due to the secretion of multiple peptides (Ferguson et al., 2008), and is closely related to the stomach. Peptidergic neurons in PVN release related transmitters after activation, and take part in the adjustment of physiological functions of the stomach by nerve conduction and neuro-endocrine systems (Benarroch, 2005). The corticotropin-releasing hormone (CRH, also known as the corticotropin-releasing factor, CRF) is a typical stress neuropeptide that is mainly distributed in the PVN. It coordinates the autonomic response of the gastrointestinal tract to stress (Czimmer and Tache, 2017).

Resolving the interactions between peripheral alterations and brain changes remains a challenging task. In recent years, optogenetic and chemogenetic techniques have provided high spatial and temporal resolutions, and have been used to target the manipulation of the activity of specific types of neurons. This provides a more comprehensive and precise understanding of the nervous system involved in regulating various organismal functions. In this study, we established a mouse model of stress-induced gastric motility disorder by using gastric dilation (GD). We dissected the functional organization of the PVN → DMV pathway, and investigated the principles of pathway acclimation in a mouse model of GD-induced gastric motility disorder *via* behavioral tests, neural circuit tracing, and electrophysiological, optogenetic, and chemogenetic techniques.

## 2. Materials and methods

### 2.1. Resting-state functional magnetic resonance imaging (rS-fMRI) trial

We recruited 24 healthy subjects for the study, of which 12 were males and 12 females. They were all 20–25 years of age, with a mean of 22.7 ± 1.9 years. The exclusion criterion for all participants was related to any contraindications to FMRI scanning. The procedures followed were in accordance with the World Medical Association’s Declaration of Helsinki and the Clinical Experimentation Ethics Committee of Anhui University of Chinese Medicine (ChiCTR2200055920).

All subjects were treated with acupuncture interventions performed at the abdomen and stomach (Cai et al., 2018) for 20 min under water-loaded GD conditions. rS-fMRI (GE 3.0T, GE Medical System, Milwaukee, Wisconsin) and gastric electromyography (Abbreviated as EGG, EGEG-2D6B type, Hefei Huake Electronic Technology Research Institute) were performed before and after acupuncture. For water-loaded GD (van Dyck et al., 2016), all subjects fasted for 6–8 h, then drank 100 ml of water within 20 s, and continued to drink pure water at about 37°C until they began to feel full. They continued to drink water in this mode until they felt completely full or could not continue owing to epigastric symptoms. We recorded the water intake at this time, that is, the maximum threshold of gastric satiety.

The Data Processing Assistant for rS-fMRI (DPARSF) 4.2 software based on the MATLAB R2013b platform and Statistical Parametric Mapping (SPM) 12 software were used to pre-process the raw data. In this study, the correlation analysis method based on the seed point region of interest (ROI) was used. By using the built-in WFU-Pick-Atlas tool in SPM12 software, two spherical seed points with a radius of 2 mm were constructed as bilateral hypothalamic ROIs based on the Montreal Neurological Institute (MNI) coordinates defined in the literature (Lips et al., 2014). The coefficients of functional connectivity between the ROI and the brain voxels were obtained by REST 1.8 software and voxel-wise analysis. The calculated values of the coefficient of functional connectivity *r* were converted into *Z*-values by Fisher’s Z, and statistically significant changes in functional connectivity were presented as images by using Alphasim correction.

### 2.2. Ethical approval and animals

We used 8–10-week-old C57BL/6J and CRH-Cre male mice, obtained from the Charles River or The Jackson Laboratory. The mice were housed in groups of five per cage in a stable environment (23–25°C, 50% humidity, 12-h light–dark cycles) for rearing, except for the mice used for surgery. After gastric balloon implantation, the mice were provided with liquid food. All experiments were conducted in accordance with the ARRIVE guidelines (Percie du Sert et al., 2020) and the Animal Experimentation Ethics Committee of Anhui University of Chinese Medicine (Reference no. AHUCM-mouse-2021-85).

### 2.3. Animal models

The mice were anesthetized with 1.5–3.0% isoflurane, and the surgical area was shaved and disinfected. Mice were fasted for 24 h prior to gastric surgery (with water provided). A catheter with a balloon (7 cm long, 0.6 mm outer diameter, 0.3 mm inner diameter) was used. A skin incision approximately 5 mm long was made at the head–neck junction on the back of the mouse, and a skin incision was made below the xiphoid cartilage at an intersection of 0.5 cm next to the midline of the abdomen. The two incisions were connected by inserting a sterile catheter. The stomach was exposed along the abdominal incision, the balloon was placed in the stomach, and then the gastric wall, abdominal muscles, and skin incisions were sutured in sequence. The other end of the catheter was passed from the abdominal incision to the neck incision, and was fixed at the neck incision with sutures. After surgery, mice were housed individually access to liquid diet. To avoid surgical stress reactions, GD and related experiments were performed 3 days after surgery.

In the GD experiment, 500 μl of 37°C saline was injected into the balloon with a rate of 0.5 mL/s for GD (McConnell et al., 2008; Liu et al., 2019; Kim et al., 2020) and lasting 20 min. Control mice receive the same gastric surgery (placement of the balloon in the stomach) as the GD group mice, but without GD. We checked the connection between the balloon and catheter at the end of the experiment. If leakage had occurred, the relevant data were excluded.

### 2.4. Animal electroacupuncture (EA) process

The site of animal electroacupuncture intervention was consistent with those used in previous experiments (Wang et al., 2015). Zhong-wan-acupoint (RN12) was located on the intersection of the upper 1/3 and lower 2/3 of the line connecting the xiphoid process and the upper border of the pubic symphysis, wei-shu-acupoint (BL21) was 2 mm adjacent to the spinous process of the 12th thoracic vertebra. Using disposable sterile acupuncture needles (0.25*13 mm, Yunlong Medical Co., Ltd., China) and electrical stimulator (G6805, Qingdao Xinsheng, China). A 20 min EA procedure was performed at a frequency of 2/100 Hz by using current with an intensity of 0.1–1 mA.

### 2.5. Virus microinjection

An intraperitoneal injection of pentobarbital (20 mg per kg) was used to induce anesthesia for stereotaxic brain injection by using a stereotactic frame (RWD Co., Ltd., China). Throughout the procedure, a heating pad was used to maintain the animals’ body temperature at 36°C.

Through calibrated glass microelectrodes connected to an infusion pump (micro4, WPI Co., Ltd., USA), 200 nl of the virus was injected (depending on the virus titer and expression strength) at a rate of 35 nl min^–1^ (unless otherwise stated). Virus overflow was prevented by leaving the pipette for a minimum of 10 min at the injection site. Three coordinates were used: anterior–posterior (AP) from the bregma, medial–lateral (ML) from the midline, and dorsal–ventral (DV) from the brain surface. All viruses used in this study were obtained from BrainVTA Co., Ltd. (Wuhan, China).

#### 2.5.1. Anterograde tracing

To allow EYFP expression in the downstream fibers, rAAV-Ef1α-DIO-hChR2 (H134R)-EYFP-WPRE (abbreviation rAAV-DIO-ChR2-EYFP, AAV2/9, 2.0E + 12 vg/mL, BrainVTA Co., Ltd) was injected into the PVN (−0.62 mm AP, ± 0.25 mm ML, −4.55 mm DV) of the CRH-Cre mice. After 3 weeks, their brain slices were co-stained with acetylcholine-specific antibodies (abbreviation ChAT) to track EYFP^+^ signals in the DMV.

#### 2.5.2. Retrograde tracing

The C57BL/6J mice were injected with scAAV2/R-hSyn-EGFP-WPREs (AAV2R, 5.09E + 12 vg/mL, BrainVTA Co., Ltd) into the DMV (−7.83 mm AP, ± 0.24 mm ML, −3.6 mm DV), this virus could be absorbed by the terminals at the injection site and transported retrogradely to the soma to express the EGFP. After 3 weeks of virus injection, the brain sections were prepared to follow the EYFP^+^ signals and co-stained with CRH-specific antibodies.

#### 2.5.3. Optogenetic manipulation

Two Cre-dependent viruses were delivered to the PVN of the CRH-Cre mice, respectively: rAAV-Ef1α-DIO-hChR2(H134R)-EYFP-WPRE-hGh-pA (abbreviation rAAV-DIO-ChR2-EYFP, AAV2/9, 2.0E + 12 vg/mL, BrainVTA Co., Ltd) and rAAV-Ef1α-DIO-eNpHR3.0-EYFP-WPRE-hGh-pA (abbreviation rAAV-DIO- eNpHR3-EYFP, AAV2/9, 2.0E + 12 vg/mL, BrainVTA Co., Ltd), rAAV-DIO-EYFP-WPRE-pA (abbreviation rAAV-DIO–EYFP, AAV2/9, 1.95 × 1,012 vgml/mL, BrainVTA Co., Ltd) viruses were used as the controls. Optical fibers (200 μm OD,0.37 NA, Inper Co., Ltd., Hangzhou, China) were embedded in the ipsilateral DMV after injection of optogenetic activation virus in the right PVN; two optical fibers (200 μm OD,0.37 NA, Inper Co., Ltd., Hangzhou, China) were embedded in the bilateral DMV after injection of optogenetic suppression virus in bilateral PVN, and fixed with dental cement.

#### 2.5.4. Chemogenetic manipulation

The Cre-dependent viruses rAAV-Ef1α-DIO-hM3d(Gq)-mCherry-WPREs (abbreviation rAAV-DIO-hM3Dq-mCherry, AAV2/9, 2.0E + 12 vg/mL, BrainVTA Co., Ltd) and rAAV-Ef1α- DIO-hM4D(Gi)-mCherry-WPREs (abbreviation rAAV-DIO- hM4Di-mCherry, AAV2/9, 2.0E + 12 vg/mL, BrainVTA Co., Ltd) were delivered to the PVN of the CRH-Cre mice, respectively. The virus rAAV-Ef1α-DIO-mCherry-WPRE-pA (abbreviation rAAV-DIO–mCherry, AAV2/8, 8.93 × 1012 vgml/mL, BrainVTA Co., Ltd) were used as controls for the chemogenetic virus (rAAV-DIO-hM4Di-mCherry/rAAV-DIO-hM3Dq-mCherry, BrainVTA Co., Ltd), and the chemogenetic virus + saline group was used as a control for the chemogenetic virus + Clozapine-N-oxide (CNO) group.

For this part of the experiment, activating viruses were injected into the right PVN and inhibiting viruses were injected into both PVNs. Confocal microscopy (LSM880, Zeiss, Germany) was used to acquire signals associated with virus injection in regions of the mouse brain.

### 2.6. *In vivo* optogenetic and chemogenetic manipulations

#### 2.6.1. Optogenetic manipulation

Optogenetic activation or inhibition experiments were performed 3 weeks after viral expression. Chronically implantable fibers were connected to a laser generator using optic fiber sleeves. A Master-8 pulse stimulator (Shanghai Fiblaser Technology Co., Ltd., China) was used to deliver a 5-min pulse of blue (473 nm, 10 Hz, 5–8 mW) or yellow light (594 nm, 5–8 mW, constant). In optogenetic inhibition experiments, yellow light was given immediately after establishing the GD model and gastric motility was recorded for 20 min; The control group was not given yellow light after establishing the GD model. In the optogenetic activation experiment, gastric motility was recorded for 20 min immediately after the application of blue light in the light group and in the absence of light in the control group; in the EA group, electroacupuncture was performed for 20 min immediately after the blue light and gastric motility was recorded at the end of the electroacupuncture treatment.

#### 2.6.2. Chemogenetic manipulations

Chemogenetic activation or inhibition experiments were performed 3 weeks after viral expression. In chemogenetic inhibition experiments, after clozapine-N-oxide (CNO, 5 mg/kg, Sigma) or saline was injected intraperitoneally for 40 min, the GD model was established and maintained 20 min, within which time (20 min) the gastric motility was detected. In chemogenetic activation experiments, after 40 min of CNO/saline injection, gastric motility was recorded (Supplementary Figure 1).

### 2.7. Optical fiber-based Ca^2+^ signal recording

A total of 200 nL of rAAV-CRH-GCaMP6s-WPRE-hGH (abbreviation rAAV-CRH-GCaMP6s, AAV2/9, 2.0E + 12 vg/mL, BrainVTA Co., Ltd) was microinjected unilaterally into the PVN of the C57BL/6J mice. An optical fiber (200 μm OD,0.37 NA, Inper) was implanted roughly 0.2 mm above the site of viral injection. 3 weeks after the mice had received the viral injection and optical fiber implantation, they were subjected to fiber photometry recording. A special balloon catheter was implanted in the stomach (refer to Method 2.3) of the mice 2 days prior to recording.

GCaMP6s fluorescence intensity was recorded before and during mechanical stimuli (GD). The values of fluorescence change Δ*F*/*F* (%) were derived by calculating Δ*F*/*F* (%) = (*F*_signal_-*F*_baseline_)/*F*_baseline_ × 100, where *F*_baseline_ is the mean of the GCaMP6s signal for 5 s before time zero (stimulus initiation), *F*_signal_ is the GCaMP6s signal for the entire session (Zhu et al., 2021). Typical Ca^2+^ traces and thermograms were generated with InperPlot software (Inper Technology). We retroactively validated the reliability of fiber optic insertion and viral infection.

### 2.8. *In vitro* electrophysiological recordings

#### 2.8.1. Brain section preparation

The pentobarbital-anaesthetized mice were intracardially perfused with 20–30 ml of ice-cold oxygenated N-methyl-d-Glucosamine artificial cerebrospinal fluid (NMDG ACSF) (solution components provided in the Supplementary Data 1). Coronal sections (300 μm) containing PVN or DMV were sectioned on a vibrating microtome (VT1200s, Leica, Germany) at a speed of 0.14 mm/s. The brain sections were first incubated in NMDG ACSF at 33°C for 12 min and then transferred to N-2-hydroxyethylpiperazine-N-2-ethanesulfonic acid (HEPES) ACSF (solution components provided in the Supplementary Data 1) at 25°C for 1 h. The brain sections were then placed in a sectioning chamber (Warner Instruments, USA) for whole-cell recording while being continuously perfused with standard ACSF (solution components in the Supplementary Data 1) at 2.5–3 ml/min at 32°C.

#### 2.8.2. Whole-cell patch-clamp recordings

Whole-cell patch-clamp recordings were performed on visualized PVN and DMV neurons using an infrared-differential interference contrast (IR/DIC) microscope (BX51WI, Olympus, Japan) with a 40x water-immersion objective. Patch pipettes (3–5 MΩ) were pulled from borosilicate glass capillaries (VitalSense Scientific Instruments Co., Ltd) using a four-stage horizontal micropipette puller (P1000, Sutter Instruments, USA), patch pipettes were filled with intracellular solution (solution components in the Supplementary Data 1) were used for voltage-clamp recording. Signals were amplified with a Multiclamp 700B amplifier, low-pass filtered at 2.8 kHz, digitized at 10 kHz, and recorded in a computer for offline analysis using Clampfit 10.7 software (Molecular Devices) (Zhou et al., 2022).

The current-evoked firing of PVN*^CRH^* neurons was recorded in current-clamp mode (*I* = 0 pA). The threshold current of the action potential was defined as the minimum current to elicit an action potential. To visualize the PVN neurons, we injected rAAV-DIO-EYFP into the CRH-Cre mice so that green fluorescent EYFP was expressed only in the CRH neurons.

For validation of chemogenetic virus function. After 3 weeks of chemogenetic virus expression, electrophysiological brain slices were prepared by the above process. The PVN neurons expressing m-Cherry were visualized by using a vertical microscope in Mercury lamp mode, and neuronal responses were recorded before and after CNO administration.

In the vitro electrophysiological recordings of light-evoked response, brain slices were prepared by the above process after 3 weeks of optogenetic virus expression, blue light was delivered through an optical fiber (diameter of 200 μm, Inper) that was positioned 0.2 mm above the surface of the target areas. To characterize the function of rAAV-DIO-ChR2-EYFP in the PVN, ChR2-EYFP^+^ neurons in PVN were visualized by a vertical microscope in Mercury lamp mode, and the responses elicited by different frequencies of blue light stimulation (473 nm, 5–8 mV, pulse width 10 Mm, stimulation frequencies 5 Hz, 10 Hz, 20 Hz) were recorded. For recording light-evoked postsynaptic currents (Fang et al., 2020; Zhou et al., 2022), DMV expressing ChR2-EYFP^+^ fibers were visualized by a vertical microscope in Mercury lamp mode. The membrane potentials were held at −70 mV for recording the excitatory postsynaptic currents and at 0 mV for recording inhibitory postsynaptic currents, and these recordings were immediately terminated once the series resistance changed more than 10%. To eliminate the polysynaptic components, tetrodotoxin (TTX; 1 μM, Dalian Refine Biochemical Items Co., Ltd.) and 4-aminopyridine (4-AP; 2 mM, Sigma) were added to the standard ACSF to block sodium channels and augment light-induced postsynaptic currents, respectively.

### 2.9. Immunohistochemistry and imaging

Mice were deeply anesthetized with an intraperitoneal injection of pentobarbital sodium and then perfused with ice-cold 0.9% saline followed by 4% PFA. The brain tissue was embedded into dehydrated paraffin and cut into 5 μm-thick sections, alternatively, coronal sections were cut to a thickness of 40 μm using a cryostat (CM1860, Leica). For immunofluorescence, the sections were incubated with blocking buffer (0.3% Triton X-100, 10% donkey serum in phosphate buffer saline) for 1 h at room temperature, and then they were treated with primary anti-bodies diluted with blocking solution, including anti-c-Fos (1:100, mouse, Santa Cruz), anti-CRH (1:500, rabbit, Abcam), anti-acetylcholine (1:500, goat, Merck), anti-GABA (1:500, rabbit, Sigma), anti-c-Fos (1:500, goat, Santa Cruz), at 4°C for 24 h. The sections were treated for 2 h at room temperature with the matching fluorophore-coupled secondary anti-body (1:500, Invitgen), or secondary anti-body sheep anti-mouse IgG (1:500, Beyotime). After rinsing, the slices were treated with 4,6-diamidino-2-phenylindole (DAPI; 1:2,000, Sigma) at the last stage. To display the fluorescent signals, the sections were photographed and scanned using the confocal microscopy (LSM880, Zeiss).

### 2.10. *In vivo* gastric recordings

Following the same surgical procedure as was performed on the model group, two incisions were made in the scapula and the abdomen, and they were connected by inserting a sterile catheter. The stomach was exposed and a custom strain gauge transducer (120 Ω, GuangCe Co., Ltd) was sticked on the surface of the gastric wall on the gastric antrum (Kawachi et al., 2011; Gao et al., 2021; Supplementary Figure 2). Another skin incision is made along the dorsal midline. The lead from the transducer is passed through the abdominal wall and extended posteriorly under the skin. Strain gauge signals were amplified by a bridge (ML-301, AD Instruments) and digitizer (Powerlab 26/04, AD Instruments) to provide automatic collection of gastric motility data. Each mouse was recorded for at least 20 min and the average amplitude and frequency were automatically calculated using Labchart 8 software (AD Instruments) with low-pass filtering and high-pass filtering. After surgery, mice were housed individually access to liquid diet.

### 2.11. Statistical analysis

The data were analyzed by investigators blind to the treatments. Owing to missing targets, such as the injection of the viruses or the positioning of the optical fiber, the viral tracing, *in vivo* recording, and behavioral data on some animals were removed from subsequent examination. The paired *t*-test was used to compare the results before and after the treatment of the same subject. For experimental groups with multiple comparisons, the data were analyzed by using one-way and two-way analysis of variance (ANOVA). Tukey’s method was used for comparisons between groups within multiple groups. The data were reported as the mean ± standard deviation and significance was defined as *p* < 0.05.

## 3. Results

### 3.1. Functional connectivity between hypothalamus and brainstem increased in subjects with water-loaded GD state following EA

**FIGURE 1:**
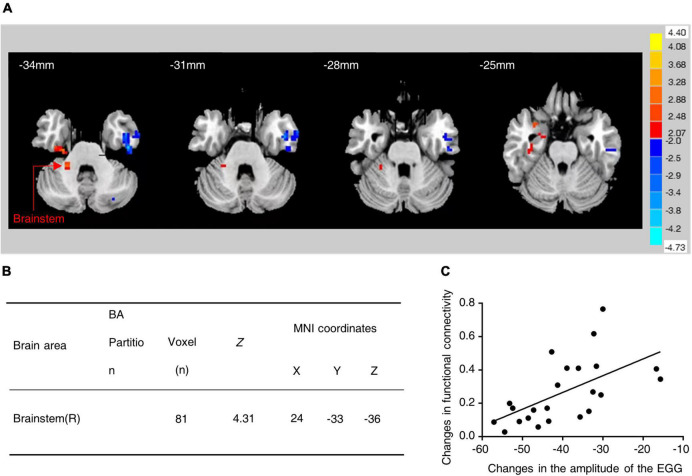
Functional connectivity between hypothalamus and brainstem was increased in subjects with water-loaded-GD state following EA. **(A)** Representative images of brain regions (brainstem) with changes in hypothalamic functional connectivity before and after EA. **(B)** The specific values of changes in hypothalamic-brainstem functional connectivity before and after EA. **(C)** EA-induced hypothalamic-brainstem (*r* = 0.583, *P* = 0.004) was positively correlated with changes in EGG amplitude, EGG, electrogastrogram.

The correlation between changes in the functional connectivity of the brain regions and changes in gastric motility was analyzed based on the seed points (regions of interest). The hypothalamus was used as the ROI and its functional connectivity with other brain regions was examined. Interestingly, it was found that the fMRI in water-loaded GD subjects showed enhanced functional connectivity between the hypothalamus and the brainstem after EA intervention (Figures 1A, B) and the changes in this functional connectivity were positively correlated with changes in EGG amplitude (*r* = 0.583, Correlation Analysis) (Figure 1C). This phenomenon suggests an association between hypothalamic–brainstem connectivity and gastric motility in water-loaded GD subjects.

### 3.2. GD increased PVN*^CRH^* neuronal activity in mice

**FIGURE 2:**
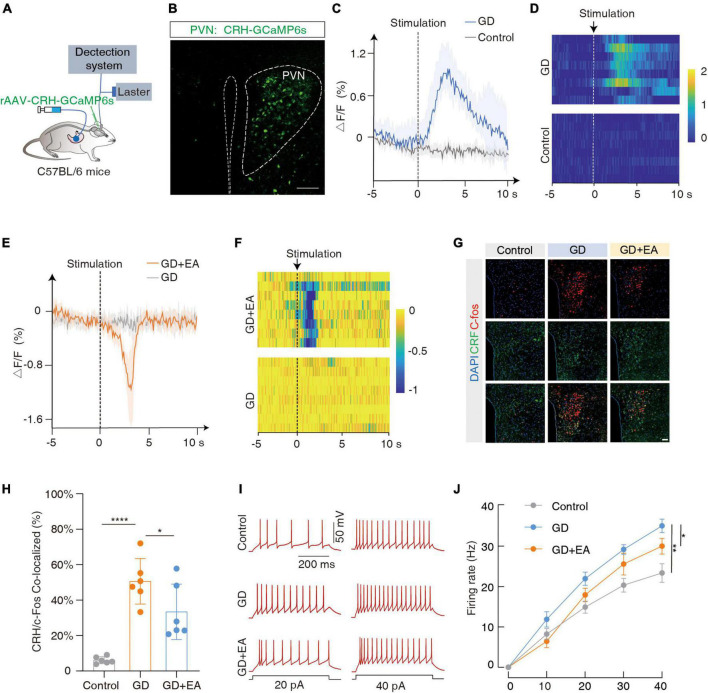
GD increases PVN^CRH^ neuronal activity in mice. **(A)** Schematic of the fiber photometry setup. Ca^2+^ transients were recorded from GCaMP6s-expressing PVN^CRH^ neurons in mice. **(B)** Typical images showing the injection site within the PVN by rAAV-CRH–GCaMP6s, scale bar 100 μm. **(C,D)** The mean (left) and the heatmaps (right) show that Ca^2+^ signals rapidly increased in gastric dilation (GD) state compared with normal state in mice. The colored bar on the right indicates ΔF/F (%). Each line in the heat map represents one experiment with one mouse. **(E,F)** The mean (left) and heat map (right) show that the Ca^2+^ signal decreases rapidly in GD mice upon acupuncture stimulation. EA is the abbreviation for acupuncture stimulation. **(G)** Representative images of C-fos and corticotropin-releasing hormone (CRH) expression in the PVN of various groups of mice. GD: gastric dilation group; EA: electroacupuncture group, scale bar 50 μm. **(H)** The C-fos and CRH co-labeling rate statistics for each group, *n* = 6 mice per group, one-way ANOVA, Control vs. GD (*t* = 6.581, *P* < 0.0001); EA vs. GD (*t* = 2.524, *P* = 0.0226). **(I,J)** Sample traces **(I)** and data **(J)** of firing rates recorded from PVN^CRH^ neurons of mice treated in control group, GD group and EA group. *n* = 10 cells from six mice per group, two-way ANOVA, Control vs. GD [*F*_(1,18)_ = 13.50, *P* = 0.0017]; EA vs. GD [*F*_(1, 18)_ = 4.703, *P* = 0.0438]. *P < 0.05, **P < 0.01, ****P < 0.0001.

The CRH neurons are mainly distributed in the PVN, and coordinate the autonomic response of the gastrointestinal tract to stress. We focused on the PVN*^CRH^* neurons (Rosenberg, 1989). To investigate whether they were sensitive to GD stimulation, we performed fiber optic photometric recordings in mice receiving the infusion of a fluorescent Ca^2+^ indicator in the PVN for the CRH promoter (rAAV-CRH-GCaMP6s) (Figures 2A, B). The Ca^2+^ signals increased rapidly following GD stimulation (Figures 2C, D). The immunofluorescence experiments (Figures 2G, H) showed an increased co-labeling rate of CRH and C-fos within PVN in the GD group compared with the normal mice (*t* = 6.581, ****P < 0.0001, one-way ANOVA). Whole-cell recordings of the PVN*^CRH^* neurons were performed in acute brain sections, and we found increased current-evoked action potentials in the GD mice [*F*_(1, 18)_ = 13.50, **P = 0.0017, two-way ANOVA]. These results suggest that the excitability of the PVN*^CRH^* neurons is enhanced in GD situations (Figures 2I, J).

Meanwhile, we found that EA intervention inhibited PVN*^CRH^* neuronal excitability in GD mice. Ca^2+^ fiber optic recording showed a rapid decrease in Ca^2+^ signal in GD mice after acupuncture stimulation (Figures 2E, F); immunofluorescence showed a decrease in co-labeling rate in the GD + EA group compared with the GD group (*t* = 2.524, *P = 0.0226, one-way ANOVA; Figures 2G, H); and membrane clamp recording showed a decrease in current-evoked action potentials in GD + EA mice compared with the GD group [*F*_(1, 18)_ = 4.703, *P = 0.0438, two-way ANOVA]. Hence, EA intervention reduces excitability of PVN*^CRH^* neurons activated by GD (Figures 2I, J).

### 3.3. Gastric motility disorders induced by GD were alleviated by inhibition of PVN*^CRH^* neuronal activity in mice

The above experiments revealed that the excitability of PVN*^CRH^* neurons was closely related to GD. We modulated the neuronal activity for further observation.

**FIGURE 3:**
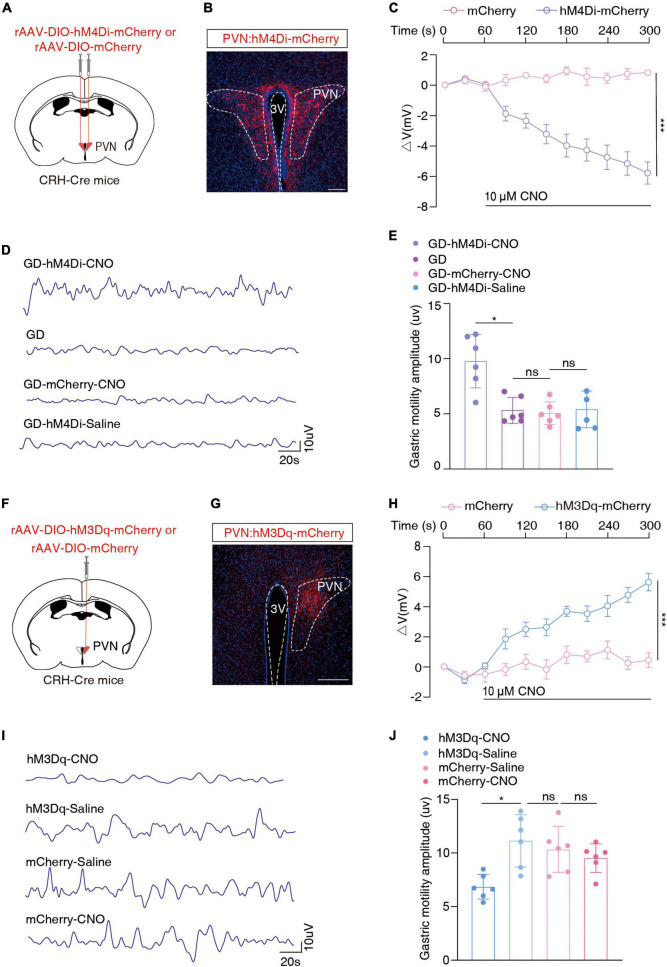
Chemogenetic inhibition of PVN^CRH^ neuronal activity alleviates GD-induced gastric motility disorders. **(A)** Schematic of chemogenetic experiments in CRH-Cre mice. **(B)** Typical images showing the injection site within the PVN by inhibition chemogenetic virus. Scale bars, 100 μm. **(C)** Membrane potential change induced by CNO, *n* = 5 cells from five mice for each group, two-way ANOVA, *F*_(1, 8)_ = 48.89, *P* = 0.0001. Action potentials induced by CNO in neurons with red fluorescence in the PVN region were recorded on brain slices containing hm4Di-mCherry and those containing mCherry, respectively. **(D)** Representative graphs of gastric motility in various groups of mice. **(E)** Effects of chemogenetic inhibition of PVN^CRH^ neurons on gastric motility in GD mice, *n* = 6 mice in each group, one-way ANOVA, *t* = 4.064, *P* = 0.0181. **(F)** Schematic of chemogenetic experiments in CRH-Cre mice. **(G)** Typical images showing the injection site within the PVN by activated chemogenetic virus. Scale bars, 100 μm. **(H)** Membrane potential change induced by CNO, *n* = 5 cells from five mice per group, two-way ANOVA, *F*_(1, 8)_ = 45.52, *P* = 0.0001. **(I)** Representative graphs of gastric motility in various groups of mice. **(J)** Effect of chemogenetic activation of PVN^CRH^ neurons on gastric motility in normal mice. *n* = 6 mice in each group, one-way ANOVA, *t* = 3.901, *P* = 0.023. *P < 0.05, ***P ≤ 0.0001.

We injected the Cre-dependent inhibitory chemogenetic virus (rAAV-DIO-hM4Di-mCherry) into the bilateral PVN of the CRH-Cre mice to selectively inhibit PVN*^CRH^* neurons (Figures 3A, B). Prior to behavioral assays, the function of the chemogenetic virus was verified by the membrane clamp of the brain slice, and its combination with CNO was effective in inhibiting PVN*^CRH^* neurons [*F*_(1, 8)_ = 48.89, ***P = 0.0001, two-way ANOVA; Figure 3C; Supplementary Figure 3A]. After 40 min of the intraperitoneal injection of CNO, the GD mice showed a significant increase in the amplitude of gastric motility (*t* = 4.064, *P = 0.0181, one-way ANOVA). However, no significant changes in this amplitude were observed in the mCherry + CNO group or the hm4Di-mCherry + saline group (Figures 3D, E). These results suggest that the inhibition of PVN*^CRH^* neuronal activity alleviates gastric motility disorders in mice under stressful conditions.

Given the increased excitability of PVN*^CRH^* neurons in the GD model mice, we injected the Cre-dependent excitatory chemogenetic virus (rAAV-DIO-hM3Dq-mCherry) into the blank CRH-Cre mice to activate PVN*^CRH^* neurons (Figures 3F, G). Prior to behavioral experiments, the function of the chemogenetic activation virus was verified, and its combination with CNO was effective in activating PVN*^CRH^* neurons [*F*_(1, 8)_ = 45.52, ***P = 0.0001, two-way ANOVA; Figure 3H; Supplementary Figure 3B]. After 40 min of intraperitoneal injection, this manipulation of activation of the PVN*^CRH^* neurons reduced the amplitude of gastric motility in the mice (*t* = 3.901, *P = 0.023, one-way ANOVA; Figures 3I, J). These results provide evidence of the functional causal relationship between the PVN*^CRH^* neurons and gastric motility.

### 3.4. Dissecting the PVN*^CRH^*-to-DMV*^ChAT^* pathway

**FIGURE 4:**
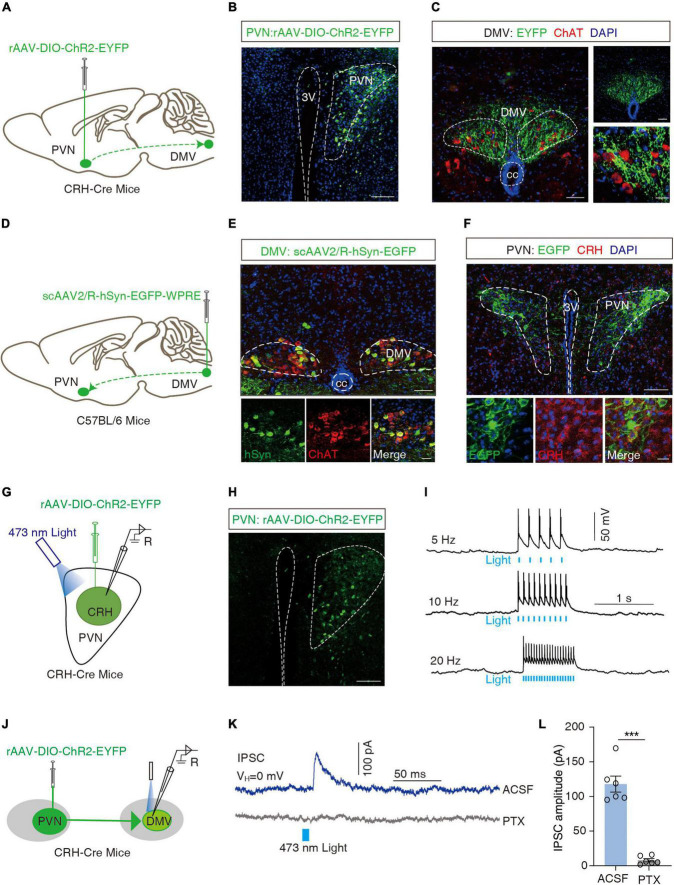
Dissection of the PVN^CRH^ to DMV^ChAT^ pathway. **(A)** Schematic of PVN injection of rAAV-DIO-ChR2-EYFP in CRH-Cre mice. **(B)** Representative image of EYFP labeling neurons by PVN infusion of rAAV-DIO-ChR2-EYFP. Scale bar, 100 μm. **(C)** Images representative of ChR2-EYFP^+^ fibers in DMV of CRH-Cre mice with PVN injection of rAAV-DIO-ChR2-EYFP (left) and ChR2-EYFP^+^ fibers co-localized with acetylcholine neuronal markers (ChAT) immunofluorescence within the DMV (right). Scale bars, 50 μm (left) or 50 μm (right-top) or 20 μm (right-bottom). **(D)** Schematic of DMV injection of scAAV2/R-hSyn-EGFP in C56BL/6 mice. **(E)** Representative image of scAAV2/R-hSyn-EGFP^+^ neurons and fibers, which co-localized with acetylcholine neuronal markers (ChAT) immunofluorescence in DMV. Scale bars, 100 μm (top) or 50 μm (bottom). **(F)** Representative image of EGFP^+^ neurons in PVN, which co-localized with CRH immunofluorescence. Scale bars, 100 μm (top) or 20 μm (bottom). **(G)** Schematic of PVN injection of rAAV-DIO-ChR2-EYFP and the recording configuration in acute slices. **(H)** Representative image injection site and viral expression within the PVN of CRH-Cre mice with PVN infusion of rAAV-DIO-ChR2-EYFP. Scale bar, 100 μm. **(I)** Sample traces of action potentials evoked by blue light (473 nm, 5–8 mV, pulse width 10 Mm, stimulation frequencies 5 Hz, 10 Hz, 20 Hz) recorded from PVN EYFP^+^ neurons in acute brain slices. **(J)** Schematic of PVN injection of rAAV-DIO-ChR2-EYFP in CRH-Cre mice and the recording configuration in acute slices. **(K)** Representative traces of light-evoked currents (473 nm, 20 ms, blue bar) before and after PTX (10 μM) treatment recorded from the DMV neurons. **(L)** Summarized data of light-evoked currents (473 nm, 20 ms) before and after PTX (10 μM) treatment recorded from the DMV neurons, *n* = 6 cells from six mice per group, paired *t*-test, *t* = 9.997, *P* = 0.0002. ***P < 0.001.

The DMV is one of the key centers of gastrointestinal regulation, and past evidence (Sawchenko, 1983; Llewellyn-Smith et al., 2012) suggests a direct fiber link between the PVN and the DMV. Moreover, more than 90% of the DMVs are cholinergic neurons (Travagli and Anselmi, 2016). To confirm the PVN*^CRH^* → DMV projection, we applied a cell type-specific anterograde tracking system and injected the anterograde rAAV-DIO-ChR2-EYFP virus into the PVN of the CRH-Cre mice (Figure 4A). 3 weeks later, neurons positive for the yellow fluorescent protein (EYFP^+^) were observed in the PVN (Figure 4B). EYFP^+^ signals were observed in the DMV, which surrounded the acetylcholine-positive (ChAT^+^) neurons with red fluorescence (Figure 4C). To further resolve the PVN*^CRH^*-to-DMV*^ChAT^* connection, we injected a broad-spectrum retrograde non-trans-synaptic virus (scAAV2/R-hSyn-EGFP) into the DMV (Figure 4D). 3 weeks later, EGFP^+^ fibers and EGFP^+^ neurons were found on the DMV, and the results of immunofluorescence showed that most of the EGFP^+^ neurons were cholinergic (red fluorescence in Figure 4E). In addition, a large number of EGFP^+^ neurons appeared in the PVN, and the results of immunofluorescence revealed multiple EGFP^+^ neurons co-labeled as CRH^+^ neurons (Figure 4F). These findings suggest a PVN*^CRH^* → DMV*^ChAT^* pathway.

To examine the functional connections of the PVN*^CRH^* → DMV*^ChAT^* pathway, optogenetic experiments were performed. We first verified the activity of rAAV-DIO-ChR2-EYFP viruses by the membrane clamp, and then recorded action potentials induced by irradiation from 473 nm blue light (5, 10, and 20 Hz) in CRH neurons expressing the ChR2-EYFP^+^ in the PVN region of the brain slice (Figures 4G–I). All CRH neurons in the PVN of the injection site were found to exhibit action potentials as induced by the blue light. As shown by the whole-cell membrane clamp combined with the optogenetic techniques, the brief stimulation of efferent fibers of ChR2-containing PVN neurons by blue light in the DMV elicited inhibitory postsynaptic currents in the DMV neurons that were eliminated by the GABA receptor antagonist picronectin [picro-toxin (PTX)] (*t* = 9.997, ***P = 0.0002, paired *t*-test; Figures 4J–L). After whole-cell membrane clamp recording, we performed immunofluorescence detection on the brain slice and found that PVN*^CRH^* neurons labeled with ChR2-EYFP were co-localized with GABAergic antibodies (Supplementary Figure 4A), and ChAT^+^ neurons in DMV were encapsulated by EYFP^+^ fibers (Supplementary Figure 4B). These data support the hypothesis that PVN*^CRH^* neurons send monosynaptic projections to DMV*^ChAT^* neurons.

### 3.5. PVN*^CRH^* neurons control the DMV*^ChAT^* neurons to alleviate gastric motility disorder induced by GD

**FIGURE 5:**
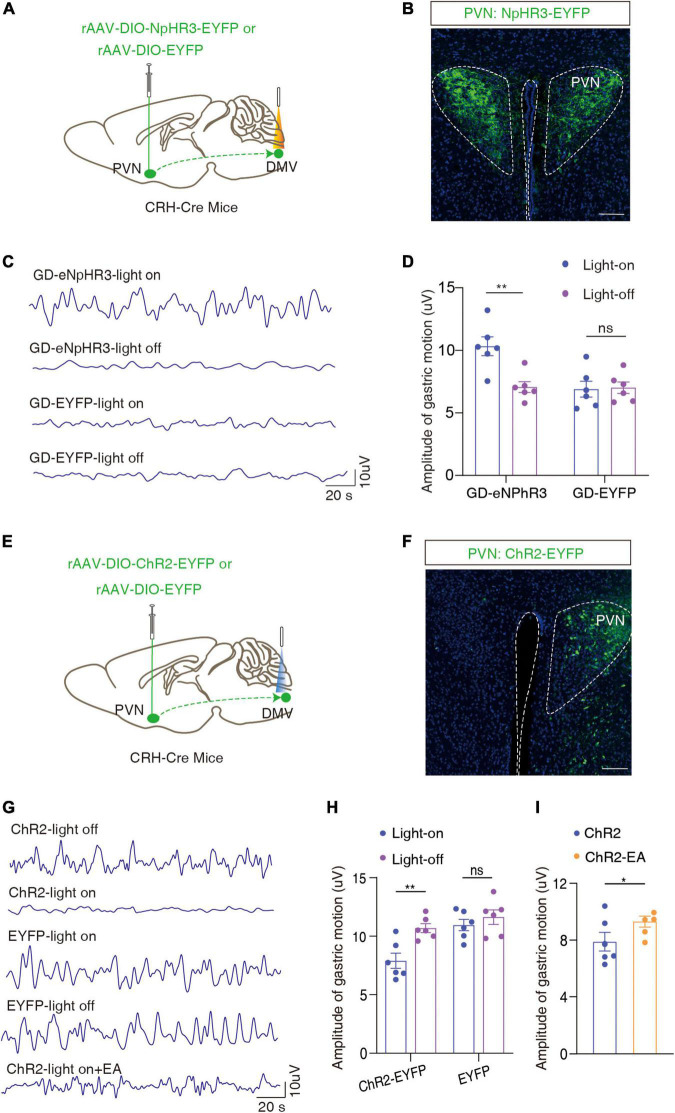
Optogenetic modulation of the PVN^CRH^ → DMV^ChAT^ pathway alleviates GD-mediated gastric motility disorders. **(A)** Schematic of optogenetic experiments in CRH-Cre mice. **(B)** Representative image of EYFP^+^ neurons by PVN infusion of rAAV-DIO-NpHR3-EYFP or rAAV-DIO-EYFP. Scale bar, 100 μm. **(C)** Representative graphs of gastric motility in various groups of mice. **(D)** Effects of optogenetic inhibition of PVN^CRH^ neurons on gastric motility in GD mice, *n* = 6 mice in each group, two-way ANOVA, *F*_1,10_ = 13.33, *P* = 0.0045. **(E)** Schematic of optogenetic experiments in CRH-Cre mice. **(F)** Representative image of EYFP^+^ neurons by PVN infusion of rAAV-DIO-ChR2-EYFP or rAAV-DIO-EYFP. Scale bar, 100 μm. **(G)** Representative graphs of gastric motility in various groups of mice. **(H)** Effects of optogenetic activation of PVN^CRH^ neurons on gastric motility in normal mice, *n* = 6 mice in each group, two-way ANOVA, *F*_(1,10)_ = 20.51, *P* = 0.0011. **(I)** Effect of EA intervention on gastric motility based on optogenetic activation of PVN^CRH^ neurons in normal mice. *n* = 6 mice in each group, paired *t*-text, *t* = 2.683, *P* = 0.0437. *P < 0.05, **P < 0.01.

To investigate the role of the PVN → DMV pathway in GD-induced gastric motility disorders, optogenetics experiments were performed. We injected the cre-dependent inhibitory optogenetic virus rAAV-DIO-eNpHR3-EYFP into the bilateral PVN and buried optical fibers in the bilateral DMV (Figures 5A, B). The optical inhibition of DMV-carrying eNpHR3 in the GD mice led to a significant increase in the amplitude of gastric motility [*F*_(1, 10)_ = 13.33, **P = 0.0045, two-way ANOVA; Figures 5C, D], which suggests that the optogenetic inhibition of the PVN*^CRH^* → DMV*^ChAT^* pathway significantly promoted gastric motility in the GD mice.

In light of the findings of the previous step, we injected Cre-dependent rAAV-Ef1a-DIO-ChR2-EYFP in naïve CRH-Cre mice and optically activated the end of the ChR2-containing PVN*^CRH^* fiber in the DMV (Figures 5E, F). We found that the activation of this pathway attenuated gastric motility in the mice [*F*_(1,10)_ = 20.51, **P = 0.0011, two-way ANOVA; Figures 5G, H]. Based on this, the amplitude of gastric motility increased after 20 min of EA intervention (*t* = 2.683, *P = 0.0437, paired *t*-text; Figures 5G, I). This result also proved, inversely, that EA may improve gastric motility by inhibiting the activity of the PVN*^CRH^* → DMV*^ChAT^* pathway. Interestingly, this result is consistent with findings from human fMRI.

## 4. Discussion

This study defined the PVN*^CRH^* → DMV*^ChAT^* pathway, which is involved in the generation of gastric motility disorders in case of GD and plays an important role in the regulation of gastric motility. Central to these processes are mechanisms of the neural pathway that involve increased excitability of PVN^CRH^ neurons and increased inhibition from them to DMV^ChAT^ neurons under acute stress-related conditions.

The PVN is a key node in the regulation of physiological stress responses, and receives multiple afferent messages about external stress and internal physiological states. It plays an important role in the regulation of gastrointestinal functions under stress. The PVN contains many stress-responsive neuron types, with a dense distribution of the CRH as a central player in the stress response (Sawchenko, 1983; Browning et al., 2014). These CRF neurons are thought to be glutamatergic or GABAergic (Tache et al., 2001). Some evidence has shown that the acute release of CRF, such as in response to a stressful event, induces plasticity within neural circuits of the vagal brainstem, which has the potential to alter the vagal output to the gastrointestinal tract (Browning et al., 2014). Moreover, pre-treatment through the injection of CRF peptide receptor antagonists into the ventricles blocks the inhibition of the gastric motor function induced by various stressors (Tache et al., 2001). The central role of the CRF in delaying gastric transit is mediated not by the stimulation of the associated HPA axis, but instead by the autonomic nervous system, as gastric inhibition of motor responses is still observed in adrenalectomized or hypophysectomized rats, but not in vagotomized rats (Lenz et al., 1988). The major structures affecting the autonomous flow to the stomach, namely, the PVN and the dorsal vagal complex (DVC) of the brain stem, were identified as the brain nuclei responsible for CRF-induced gastric emptying and motor inhibition in rats (Monnikes et al., 1992). In line with previous reports, we confirmed that GD mice exhibited significant deficits in gastric motility with activation of CRH neurons in the PVN.

The DMV is the origin of vagal efferent fibers that regulate gastric motility and other visceral functions (e.g., the vagal circuit and its effect on gastric motility) (Travagli and Anselmi, 2016). The efferent fibers of the DMV form synapses with postganglionic neurons located in the stomach to regulate gastric motility (Travagli and Anselmi, 2016), and the DMV has a direct fiber connection to the gastrointestinal tract. The vast majority of neurons in the DMV are cholinergic, and activate nicotinic cholinergic receptors on postganglionic neurons within the target organ (Browning and Travagli, 2010). There is a well-known projection between the PVN and the DMV (Willett et al., 1987), and we verified this result with anterograde and retrograde monosynaptic tracing. In line with previous reports (Browning et al., 2014), the CRF increased inhibitory GABAergic synaptic transmission to the identified corpus-projecting DMV neurons. In this study, we observed that subjects with water-loaded GD state following EA showed enhanced functional connectivity between hypothalamus and brainstem in fMRI, and the change in this functional connectivity was positively correlated with the change in EGG amplitude, then we mapped the PVN → DMV pathway in animal experiments, found through optogenetic techniques that PVN^CRH^ neurons projecting to acetylcholine neurons in the DMV were inhibitory, and alleviated gastric motility disorders in GD mice by inhibiting PVN*^CRH^* → DMV*^ChAT^* excitability.

Owing to the complex and multifactorial pathophysiology of FGIDs, the effectiveness of current treatments is still unsatisfactory. In this case, nearly 50% of FGIDs patients have exhibited a tendency to seek complementary and alternative medicine (Lahner et al., 2013). Many clinical studies and evidence-based evaluations have shown that acupuncture treatment alleviates the symptoms of FGIDs (Yuan-yuan et al., 2019; Guo et al., 2020; Wang et al., 2021) and mitigates symptoms and anxiety in FD patients. The central nervous system is an important site for the integration of acupuncture information and disease-related information. Acupuncture treatment involves the insertion of fine needles into the skin and underlying muscle layers, where the methods of stimulation may be manual or electric. Many previous studies (Koizumi et al., 1980; Kagitani et al., 2005, 2010; Takahashi, 2013) have reported that acupuncture stimulates somatic afferent nerves in the skin and muscles, when the mechanical force generated by acupuncture directly or indirectly acts on the acupoint area. Mechanical stimulation is transformed into neurochemical signals that induce afferent signals from the body. Many experiments have shown that the effect of acupuncture may be achieved by somatosensory autonomic reflexes or the modulation of the nervous system (Yu, 2020; Liu et al., 2021). The neural mechanisms involved in the modulation of gastrointestinal movement by acupuncture feature several aspects of acupuncture signaling, the sympathetic and parasympathetic nervous systems, the enteric nervous system, and the central nervous system (Wang et al., 2021). With the development of neuroimaging technology, the study of the acupuncture effect is not limited to animal experiments, non-invasive and high spatial and temporal resolution techniques, such as fMRI, support acupuncture effect based on human beings. Several neuroimaging studies have shown that acupuncture treatment improves not only clinical symptoms (postprandial fullness, epigastric distention, etc.) but also significantly modulates abnormal brain functions such as medial prefrontal cortex, brainstem, thalamus, caudate, and hippocampus in FD patients (Zeng et al., 2015; Teng et al., 2022; Yin et al., 2022). The central neural mechanism of the acupuncture effect is closely related to its modulation of neural circuits or neural networks, our study found after EA intervention, fMRI in GD subjects showed changes in functional connectivity between hypothalamus and brainstem. It also reflects the central nervous system provides exogenous neural input to control gastrointestinal motility in a broader and more integrated manner involving the spinal cord, medulla oblongata, thalamus, and so on.

The modulatory effects of acupuncture on gastrointestinal motility require the involvement of the CNS by altering the activity of nuclei associated with gastrointestinal motility, including the DMV, the NTS, the nucleus of the middle suture, the lateral hypothalamic area (LHA), and the PVN. All of these have been identified following the injection of neuro-anatomical tracers into the stomach and ST36 (Lee et al., 2001; Wang et al., 2013). The NTS and DMN form the main neuro-anatomical structure of the vagus nerve, the DVC, the role of which in acupuncture-mediated regulation of gastrointestinal function is supported by multiple pieces of evidence from several studies. The electro-acupuncture points ST36 and ST37 modulate the electrical activity of the stomach while regulating the firing of NTS and DMV neurons (Liu et al., 2004; Wang et al., 2007; Gao et al., 2012). The PVN is particularly important in the regulation of the gastrointestinal function, especially in case of stress-induced changes in gastrointestinal dynamics. Our previous study (Wang et al., 2015) found that the RN12 and BL21 signals of electroacupuncture converge in the PVN, and increase the expression of gastrointestinal hormones as well as their receptors in the PVN and the gastric antrum. Previous studies (Zhao et al., 2021) have suggested that the improvement in stress-induced jejunal motility disorders by the EA of ST36 may be related to the deregulation of CRF-R_2_. However, information on the acupuncture-mediated regulation of gastric motility by the CRH function is scarce. In this study, we found that EA modulates gastric motility by inhibiting the excitability of PVN^CRH^ neurons in GD mice. We used viral tracking nuclear electrophysiology to identify an inhibitory neural circuit of the PVN*^CRH^* → DMV*^ChAT^* pathway. Following this, the activity of this circuit was modulated by using optogenetics, and the results suggested that EA possibly improves gastric motility by inhibiting the activity of the PVN*^CRH^* → DMV*^ChAT^* pathway. This result also proved, inversely, that EA may improve gastric motility by inhibiting the activity of the PVN*^CRH^* → DMV*^ChAT^* pathway. Interestingly, this result is consistent with findings from human fMRI.

In summary, this study explored the significance of the PVN*^CRH^* → DMV*^ChAT^* pathway in GD-induced gastric motility disorders. We found that the alleviation of its symptoms through the inhibition of the pathway may involve a hypothalamic paraventricular nucleus-mediated system of autonomic control. As options for the pharmacological treatment for functional gastric motility disorders remain limited, these findings suggest the potential for non-pharmacological therapeutic approaches, and provide new insights into the mechanisms by which somatic stimulation modulates the physiological function of internal organs and systems.

## Data availability statement

The raw data supporting the conclusions of this article will be made available by the authors, without undue reservation.

## Ethics statement

The studies involving human participants were reviewed and approved by Clinical Experimentation Ethics Committee of Anhui University of Chinese Medicine. The patients/participants provided their written informed consent to participate in this study. This animal study was reviewed and approved by Animal Experimentation Ethics Committee of Anhui University of Chinese Medicine.

## Author contributions

G-MS and X-YW: conceptualization. X-YW, R-LC, and HW: methodology and formal analysis. X-YW, G-QW, and X-QC: software, validation, and writing—original draft preparation. X-YW, G-QW, SH, HW, and X-QP: investigation. R-LC, H-TW, and G-MS: resources. X-YW, G-QW, X-QC, X-QP, and H-TW: data curation. R-LC, HW, and G-MS: writing—review and editing. X-QP, M-TZ, and H-TW: visualization. G-MS: supervision and project administration. G-MS, M-TZ, and X-YW: funding acquisition. All authors contributed to the article and approved the submitted version.
