# Supplementary material for: A neural circuit for gastric motility disorders driven by gastric dilation in mice
Source: Front Neurosci. 2023 Feb 22;17:1069198. doi: 10.3389/fnins.2023.1069198 (PMC9992744; doi:10.3389/fnins.2023.1069198)
Supplement: Supplementary file 1 [file Data_Sheet_1.docx]

Supplementary Material

# Supplementary Data 1

**N-methyl-d-Glucosamine artificial cerebrospinal fluid (NMDG ACSF)** that contained (in mM) 93 N-methyl-d-glucamine (NMDG), 2.5 KCl, 1.2 NaH_2_PO_4_, 30 NaHCO_3_, 20 HEPES, 25 glucose, 2 thiourea, 5 Na-ascorbate, 3 Na-pyruvate, 0.5 CaCl_2_, 10 MgSO_4_ and 3 glutathione (GSH), pH 7.3–7.4, osmolarity was 300–305 mOsmkg^−1^.

**N-2-hydroxyethylpiperazine-N-2-ethanesulfonic acid (HEPES) ACSF** that contained (in mM) 92 NaCl, 2.5 KCl, 1.2 NaH_2_PO_4_, 30 NaHCO_3_, 20 HEPES, 25 glucose, 2 thiourea, 5 Na-ascorbate, 3 Na-pyruvate, 2 CaCl_2_, 2 MgSO_4_ and 3 GSH, pH 7.3–7.4, osmolarity was 300–305m Osmkg^−1^.

**Standard ACSF** that contained (in mM) 129 NaCl, 2.4 CaCl_2_, 3 KCl, 1.3 MgSO_4_, 20 NaHCO_3_, 1.2 KH_2_PO_4_ and 10 glucose, pH 7.3–7.4, osmolarity was 300–305 mOsmkg^−1^.

**Patch pipettes filled with intracellular solution** containing 10 mM HEPES, 5 mM KCl, 130 mM K-gluconate, 0.6 mM EGTA, 2 mM MgCl_2_, 2 mM Mg-ATP, and 0.3 mM Na-GTP, pH 7.2, osmolarity was 285–290 mOsmkg^−1^.

# Supplementary Figures

## Supplementary Figure1


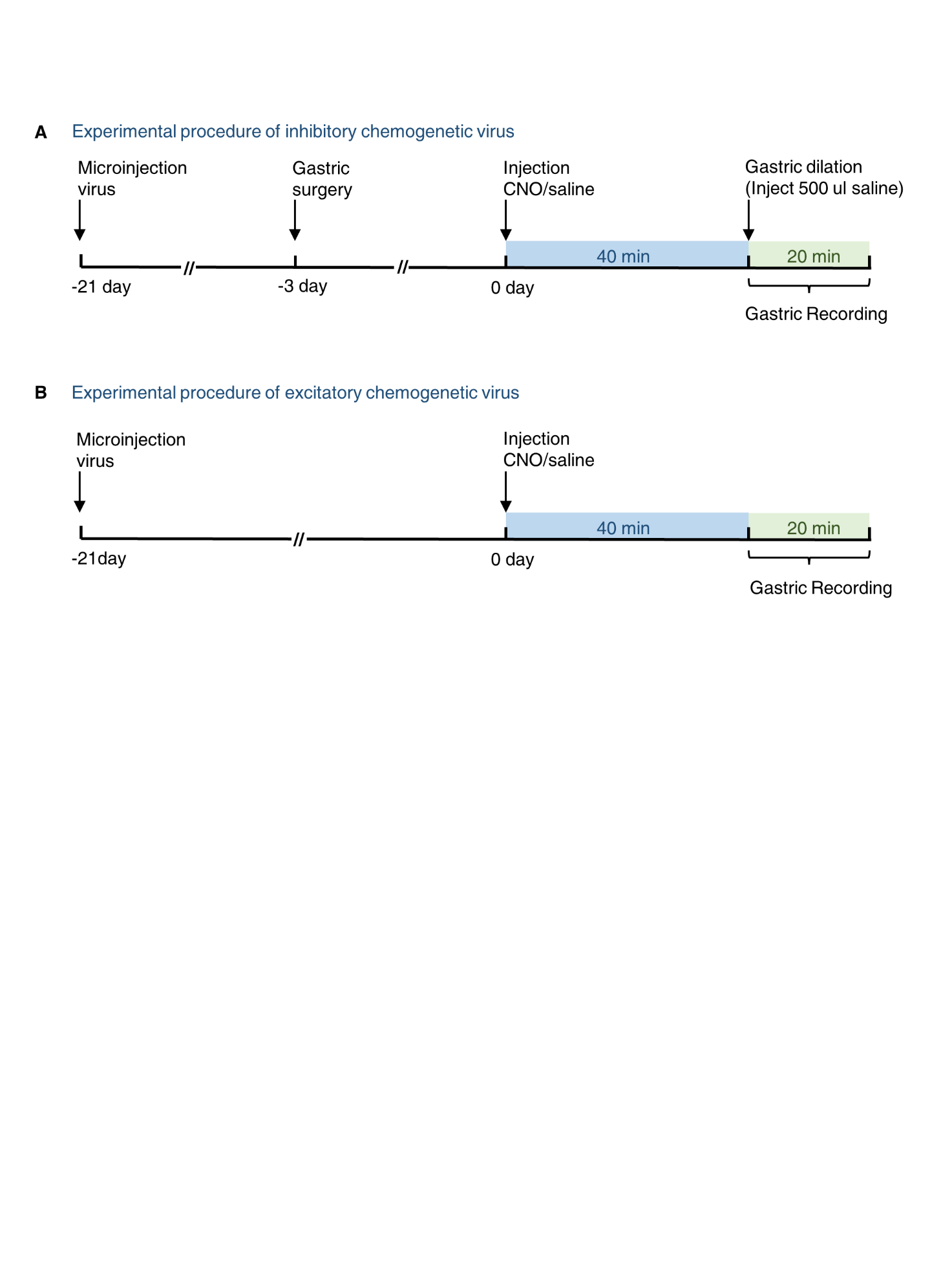


**Supplementary Figure 1. Experimental procedure of chemogenetic virus.**

**A,** Experimental procedure of inhibitory chemogenetic virus. **B,** Experimental procedure of excitatory chemogenetic virus.

## Supplementary Figure 2


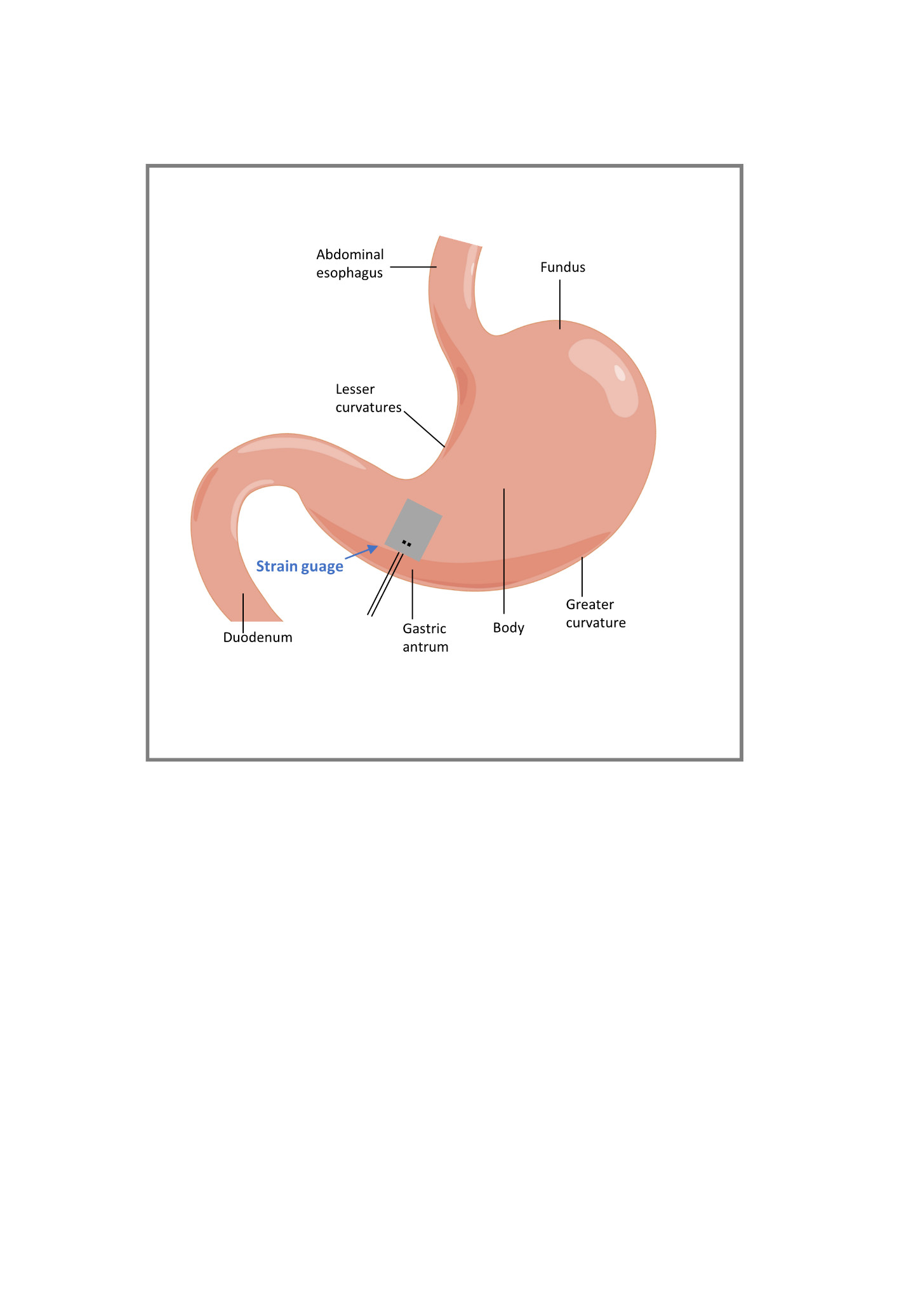


**Supplementary Figure 2.** **Schematic diagram of gastric recordings**

## Supplementary Figure 3


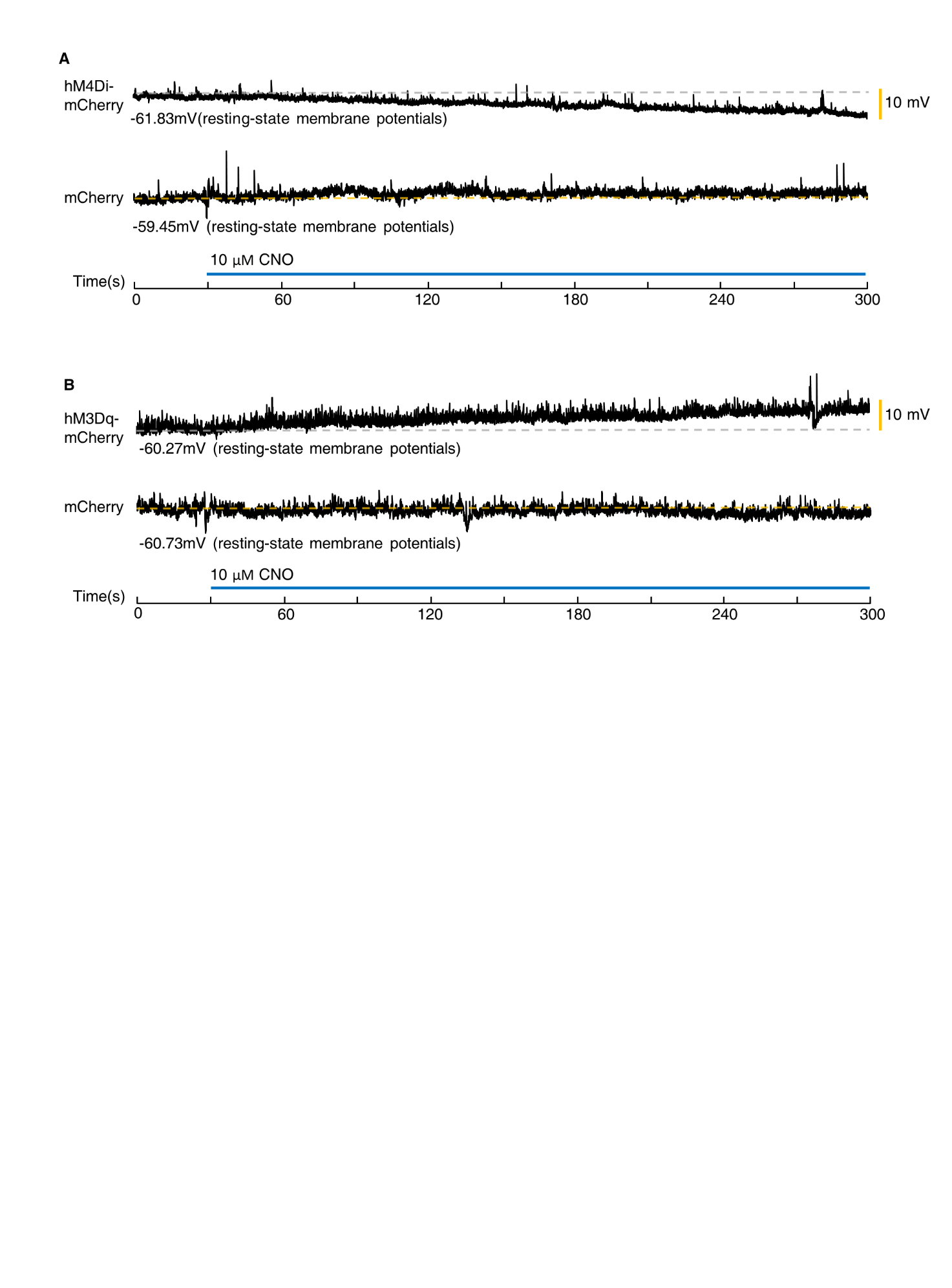


**Supplementary Figure 3. Validation of chemogenetic virus potency.**

**A,** The raw traces of whole-cell recordings at each time point showing the effect of CNO on PVN^CRH^ neurons expressing rAAV-DIO-hM4Di-mCherry or rAAV-DIO-mCherry, n = 5 cells from 5 mice for each group. Action potentials induced by CNO in neurons with red fluorescence in the PVN region were recorded on brain slices containing hM4Di-mCherry and those containing mCherry, respectively. **B,** The raw traces of whole-cell recordings at each time point showing the effect of CNO on PVN^CRH^ neurons expressing rAAV-DIO-hM3Dq-mCherry or rAAV-DIO-mCherry, n = 5 cells from 5 mice for each group. Action potentials induced by CNO in neurons with red fluorescence in the PVN region were recorded on brain slices containing hM3Dq-mCherry and those containing mCherry, respectively.

##
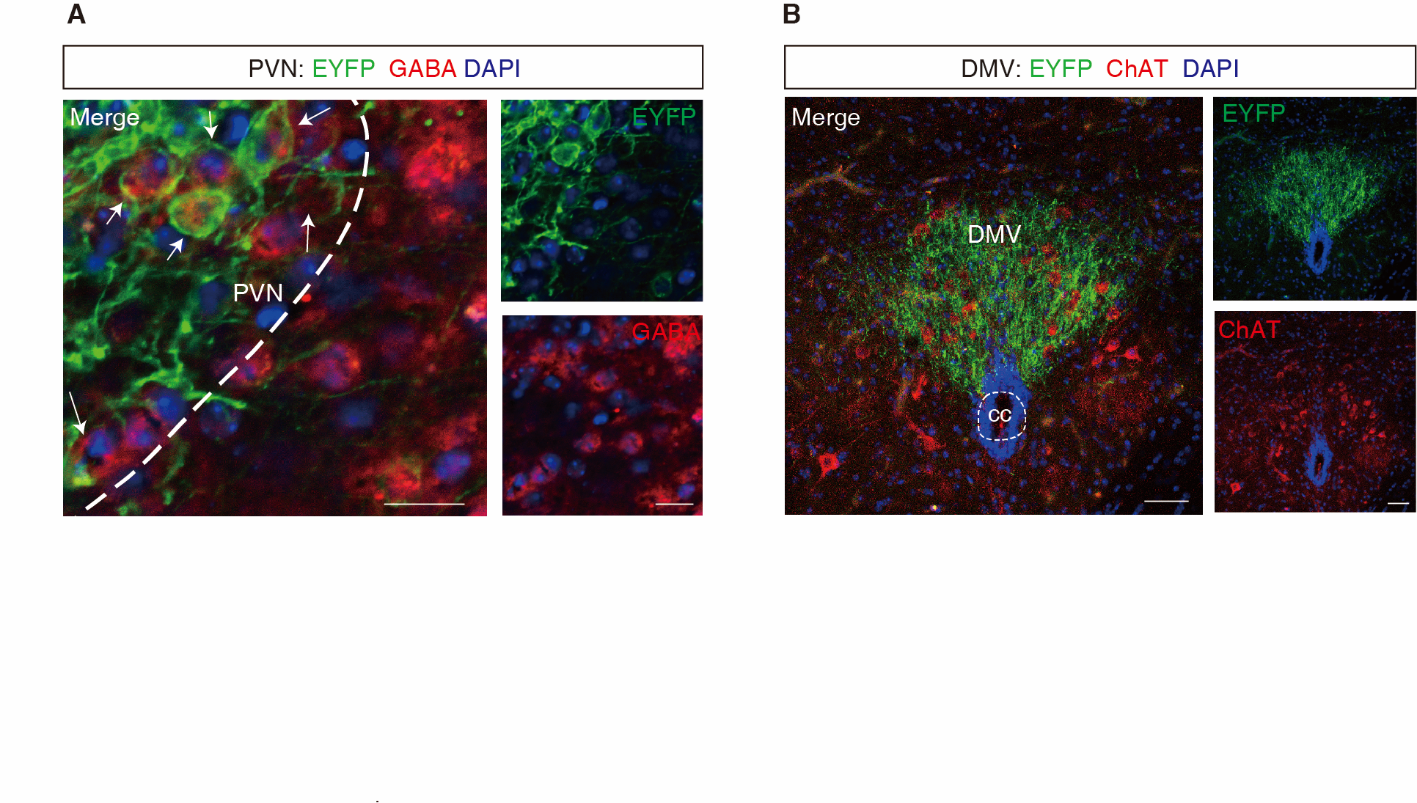
Supplementary Figure 4

**Supplementary Figure 4. Dissection of the PVN^CRH^ to DMV^ChAT^ pathway.**

**A**, Representative images of viral expression within the PVN of CRH-Cre mice infused with rAAV-DIO-ChR2-EYFP, co-localized with GABA-specific antibodies. Scale bar, 20 µm. **B**, Representative immunofluorescence images of EYFP-expressing projection fibers co-labeled with ChAT^+^ neurons in DMV. Scale bar, 20 µm.
